# Identification and Sorting of Adipose Inflammatory and Metabolically Activated Macrophages in Diet-Induced Obesity

**DOI:** 10.21769/BioProtoc.5479

**Published:** 2025-10-20

**Authors:** Dan Wu, Komal Rawal, Venkateswararao Eeda, Hui-Ying Lim, Weidong Wang

**Affiliations:** 1 Department of Genetics, Heersink School of Medicine, UAB Comprehensive Diabetes Center, University of Alabama at Birmingham, 1918 University Blvd, Birmingham, AL, USA; 2 Department of Medicine, Division of Endocrinology, The University of Oklahoma Health Science Center, 941 Stanton L. Young Boulevard, Oklahoma City, OK, USA; 3 Department of Physiology, Harold Hamm Diabetes Center, The University of Oklahoma Health Science Center, 941 Stanton L. Young Boulevard, Oklahoma City, OK, USA

**Keywords:** SVF, Adipose tissue macrophages, Flow cytometry, Obesity, Metabolism, Inflammation, IRE1 inhibition, Mouse model, eWAT

## Abstract

Adipose tissue macrophages (ATMs) critically influence obesity-induced inflammation and metabolic dysfunction. Recent studies identified distinct ATM subsets characterized by markers such as CD11c, CD9, and Trem2, associated with pro-inflammatory and metabolically activated states. This protocol outlines a detailed, reproducible methodology for isolating, characterizing, and sorting these ATM subsets from murine epididymal white adipose tissue (eWAT) using multicolor flow cytometry. Key steps include stromal vascular fraction (SVF) isolation, immunophenotyping, sequential gating strategies, and fluorescence-activated cell sorting (FACS) for downstream gene expression analysis. The protocol was validated in diet-induced obese (DIO) mice treated with the IRE1 RNase inhibitor STF-083010, demonstrating its utility for studying ATMs in the context of obesity and metabolic disease.

Key features

• Detailed isolation and identification of multiple ATM subsets from eWAT.

• Compatible with comprehensive flow cytometric analyses and fluorescence-activated cell sorting (FACS).

• Facilitates downstream gene expression profiling from sorted ATM subsets.

• Validated using a metabolic intervention (IRE1 RNase inhibitor STF-083010) in a mouse obesity model.

## Graphical overview


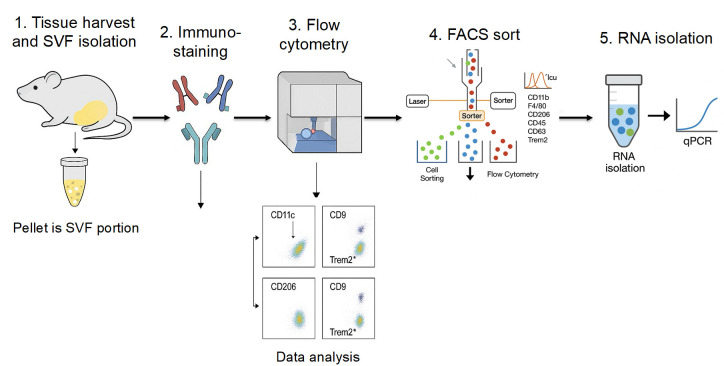


## Background

Obesity is characterized by a state of chronic, low-grade inflammation. However, excessive fatty acid release may worsen adipose tissue inflammation and contribute to insulin resistance. Obesity-induced insulin resistance is closely associated with adipose tissue inflammation, primarily driven by adipose tissue macrophages (ATMs) [1,2]. Insulin resistance precedes the accumulation of pro-inflammatory (M1) macrophages in epididymal white adipose tissue (eWAT) during diet-induced obesity in mice [3]. Some evidence showed that enhanced insulin signaling in adipocytes can protect against ATM infiltration during obesity. For example, it was demonstrated that mice with adipocyte-specific deletion of PTEN, thereby enhancing insulin signaling, were protected from ATM accumulation despite gaining weight on a high-fat diet [4]. Similarly, impaired insulin signaling via phosphoinositide 3-kinase contributes to ATM recruitment in diet-induced obesity [5]. Pro-inflammatory cytokines produced by ATMs, such as TNF-α, IL-6, MCP-1, and FABP4, exacerbate insulin resistance and adipose inflammation by activating pathways like JNK and NF-κB, creating a vicious cycle. These findings suggest that insulin resistance likely develops early in diet-induced obesity, independent of ATM infiltration, but can later drive ATM recruitment alongside other obesity-related stress responses.

Accumulating ATMs undergo a phenotypic shift from an anti-inflammatory M2 state to a pro-inflammatory M1 state, thereby amplifying both local adipose tissue and systemic inflammation through cytokine secretion [6]. However, the traditional M1/M2 macrophage classification—which distinguishes macrophages as either classically activated M1 (pro-inflammatory) or alternatively activated M2 (anti-inflammatory and tissue-repairing)—is increasingly recognized as insufficient. This binary framework does not fully reflect the spectrum of macrophage phenotypes in complex metabolic settings [7–9]. More recent studies have revealed that obesity gives rise to a dominant population of ATMs exhibiting a metabolically activated phenotype, characterized by heightened lipid handling and altered metabolic programming, which cannot be adequately explained by the conventional M1/M2 paradigm [7,10,11]. Single-cell analyses reveal distinct macrophage subsets, notably CD9^+^ and Trem2^+^ populations that are characterized as metabolically active, meaning they exhibit enhanced lipid uptake, lysosomal activity, and metabolic gene expression typical of obesity-associated ATMs. CD9 and Trem2 themselves function as surface markers rather than metabolic enzymes; their expression correlates with, but does not directly drive, the altered metabolic programming of these macrophages [8,9]. However, the relationship between the previously identified “M1-like” CD11c+ ATMs and the new CD9+ and/or Trem2+ ATM subpopulations is unclear. A potential approach to solve this would be to combine traditional protocols (e.g., CD11c-based flow cytometry) with single-cell or multiparameter analyses incorporating CD9 and Trem2, thereby enabling direct comparison of marker co-expression and metabolic function within the same macrophage populations. The role of IRE1α in adipose tissue remodeling and insulin resistance has also been demonstrated through its activity in ATMs. Recent studies show that myeloid-specific deletion of IRE1α, including in macrophages, protects mice from diet-induced obesity and insulin resistance [12].

The stromal vascular fraction (SVF) is a heterogeneous population of cells within adipose tissue, typically isolated through enzymatic digestion with agents such as collagenase. The stromal vascular fraction (SVF), which contains a heterogeneous population of cells including immune cells, endothelial cells, and preadipocytes, is typically isolated from adipose tissue through enzymatic digestion with collagenase. SVF derived from epididymal white adipose tissue (eWAT) has been widely used to study obesity-associated macrophage infiltration and inflammation [2,13]. Notably, the cellular composition of the SVF differs across adipose depots, with visceral adipose tissue such as eWAT generally harboring a higher proportion of pro-inflammatory immune cells compared to subcutaneous adipose tissue [14,15]. In addition to eWAT, which is a visceral fat depot, the inguinal white adipose tissue (iWAT) is a major subcutaneous depot often used as a comparative tissue, as it differs in cellular composition and inflammatory profile. Following the removal of mature adipocytes, connective tissue, and blood from the lipoaspirate, the remaining SVF includes a diverse mix of cell types such as mesenchymal stem cells, endothelial progenitor cells, regulatory T cells, macrophages, smooth muscle cells, pericytes, and preadipocytes. This protocol provides a systematic approach for the detailed characterization and sorting of these ATM subsets from eWAT, enabling subsequent functional and gene expression studies.

## Materials and reagents

**Biological materials**

1. C57BL/6J male mice (Jackson Laboratory, stock no. 000664)

**Reagents**

1. Collagenase P (Roche, catalog number: 11213857001)

2. Phosphate buffer saline (PBS) (Fisher Scientific, catalog number: C10010500BT)

3. Gibco^TM^ RPMI 1640 medium (Fisher Scientific, catalog number: 11875093)

4. Gibco^TM^ DMEM medium (Fisher Scientific, catalog number: 15303601)

4. Fetal bovine serum (FBS) (Gibco, catalog number: 10082147)

5. BSA solution (Solarbio, catalog number: H1130)

6. Penicillin-Streptomycin (pen/strep) (5,000 U/mL) (Gibco, catalog number: 15140122)

7. 1× PBS (Thermo Fisher, catalog number: 10010023)

8. 10× red blood cell (RBC) lysis buffer (Sigma, catalog number: R7757)

9. STF-083010 (Calbiochem/Millipore sigma, catalog number: 516535)

10. Fc block (BD Biosciences, catalog number: 563019)

11. DMSO solution (Millipore sigma, catalog number: 135623-5G)

12. E.Z.N.A. Total RNA kit (Omega Bio-Tek, catalog number: R6812-00)

13. Oligo d(T)18 mRNA primer (New England Biosystems, catalog number: S1316S)

14. SuperScript IV Reverse Transcription kit (Applied Biosystems, catalog number: 18090050)

15. PowerUp^TM^ SYBR^TM^ Green Master Mix (Applied Biosystems, catalog number: A25742)

16. Anti-mouse CD45 PerCP (BioLegend, catalog number: 110725)

17. Anti-mouse F4/80 FITC (BioLegend, catalog number: 123108, stock: 0.5 mg/mL)

18. Anti-mouse CD11b PE (BioLegend, catalog number: 101207, stock: 0.2 mg/mL)

19. Anti-mouse CD11c BV421 (BioLegend, catalog number: 117330, stock: 0.2 mg/mL)

20. Anti-mouse CD206 Alexa Fluor 700 (BioLegend, catalog number: 141734, stock: 0.5 mg/mL)

21. Anti-mouse CD9 PE-Dazzle594 (BioLegend, catalog number: 124821, stock: 0.2 mg/mL)

22. Anti-mouse CD63 PE-Cy7 (BioLegend, catalog number: 143909, stock: 0.2 mg/mL)

23. Anti-mouse Trem2 APC (R&D Systems, catalog number: FAB17291N)

25. Ethyl alcohol (Millipore sigma, catalog number: 64-17-5)

**Solutions**

1. 0.45 mg/mL Collagenase P solution (see Recipes)

2. 1 M CaCl_2_ solution (see Recipes)

3. 1× RBC lysis buffer solution (see Recipes)

4. FACS buffer (see Recipes)

**Recipes**

**1. 0.45 mg/mL Collagenase P solution**

**Table:** 

Reagent	Final concentration	Quantity or volume
Collagenase P	0.45 mg/mL	0.45 mg
DMEM	99.5%	20 mL
CaCl_2_	2 mM	100 μL

**2. 1 M CaCl_2_ stock solution**

**Table:** 

Reagent	Final concentration	Quantity or volume
H_2_O	100%	15 mL
CaCl_2_	1M	1.655 g

**3. 1× RBC lysis buffer**

**Table:** 

Reagent	Final concentration	Quantity or volume
H_2_O	90%	9 mL
10× RBC lysis buffer	1×	1 mL

a. Take 1 mL of 10× RBC lysis buffer and add 9 mL of ddH_2_O to make 1× buffer concentration.

b. Use room temperature–equilibrated RBC lysis buffer during the RBC lysis step.

**4. FACS buffer**

**Table:** 

Reagent	Final concentration	Quantity or volume
PBS	98.5%	50 mL
FBS	1.5%	750 μL

Take 50 mL of PBS and add 750 μL of FBS (1.5%). Store at 4 °C.

**Laboratory supplies**

1. 1.5 mL MaxyClear Snaplock (Axygen, catalog number: MCT-150-C)

2. 100 × 10 mm Petri dish with cover (Corning, catalog number: 70165-100)

3. Falcon^TM^ 50 mL high-clarity conical centrifuge tubes (Fisher Scientific, catalog number: 14-432-22)

4. Falcon^TM^ cell strainers 70 μm (Fisher Scientific, catalog number: 08-771-2)

5. Micro-dissecting scissors (Sigma-Aldrich, catalog number: S3146)

6. Surgical design splinter tweezer forceps (Fisher Scientific, catalog number: 22-079-762)

7. 15 mL conical tubes (Labselect, catalog number: CT-012-15)

8. 50 mL conical tubes (Labselect, catalog number: CT-012-50)

9. 10 mL serological pipettes (Corning, catalog number: 4488)

10. 25 mL serological pipettes (Corning, catalog number: 4489)

11. 10 μL pipette tips (Rainin, catalog number: 30389175)

12. 200 µL pipette tips (Rainin, catalog number: 30389191)

13. 1,000 µL pipette tips (Rainin, catalog number: 30389164)

14. Millex-GP syringe filter unit, pore 0.22 µm (Millipore, catalog number: SLGPR33RB)

15. Syringe PP/PE without needle (Millipore, catalog number: Z683604-100EA)

16. Cell culture multi-well plates (12-well plate) (Corning, catalog number: 3513)

17. Cell culture multi-well plates (6-well plate) (Corning, catalog number: 3471)

18. Countess^TM^ cell counting chamber slides (Invitrogen, catalog number: C10283)

19. Falcon^®^ round-bottom test tubes (Corning, catalog number: CLS352057)

20. Precision balances (Kern EMB, catalog number: Z742801-1EA)

## Equipment

1. Fixed-angle general-purpose centrifuge (VWR, model: 76019-132)

2. Mega star 1.6 general-purpose 1.6 L benchtop centrifuge (VWR, model: 76519-280)

3. Refrigerated centrifuge (Eppendorf, catalog number/model: 5804/5804R)

4. Corning^®^ LSE^TM^ benchtop shaking incubator with platform (Millipore Sigma, model: CLS6790)

5. Spectral flow cytometer (Cytek, catalog number/model: Aurora)

6. Cell sorter (Beckman Coulter, catalog number/model: MoFloXDP)

7. CFX96 Touch Real-Time PCR detection system (Bio-Rad, model: BIO-CFX96T)

8. NordicSafe^®^ Class II biological safety cabinet (ESCO, model: NC2-L)

9. Thermo Scientific Steri Cycle 370 CO2 Incubator (MarshallCare, model: TH-370NDS)

10. Olympus inverted fluorescence microscope (objectives: 4×, 10×, 20×) (Olympus, model: IX73)

11. Eppendorf 5810R refrigerated centrifuge w/ selectable rotor (MarshallCare, model: EP-5810R8)

12. Countess^TM^ II FL automated cell counter (Thermo Fisher Scientific, model: AMQAF1000)

13. 0.5–10 μL pipetter (Rainin, catalog number: 17014388)

14. 10–100 μL pipetter (Rainin, catalog number: 17014384)

15. 100–1000 μL pipetter (Rainin, catalog number: 17014382)

16. Racks

17. Liquid nitrogen (N2) tank

18. Freezer (-80 °C)

19. Refrigerator (2–8 °C)

## Software and datasets

1. FlowJo software (v10)

## Procedure

**A. Tissue collection and SVF preparation**

1. Clean mice thoroughly with 70% ethanol and euthanize using an approved method. After euthanasia, clean the carcass thoroughly with 70% ethanol to reduce contamination. Immediately dissect the eWAT, which is one of the largest visceral fat depots in mice and a primary site for isolating ATMs. eWAT is located adjacent to the testes in males and the uterine horns in females. Using fine scissors and blunt forceps, gently separate the fat pad from surrounding connective tissue and vasculature, taking care to avoid contamination from nearby lymph nodes or blood vessels. (The anatomical location of eWAT has been well described and illustrated in prior studies [2], which may serve as a reference for investigators less familiar with this adipose depot.)

2. Optional blood clearance (recommended for flow cytometry/scRNA-seq/proteomics): Perform cardiac perfusion with a 10 mL syringe (10 mL × 3 times) and put three small cuts in the liver lobes to flush out the maximum possible blood from the body. Wash with PBS to remove all the blood clots deposited on the tissue (This step is optional and can be omitted as RBC lysis is effectively optimized).

**Rationale**: Perfusion decreases carryover of circulating leukocytes and RBCs, improving ATM purity and reducing (a) non-ATM CD45^+^ contamination that complicates gating, (b) hemoglobin/platelet interference with collagenase digestion and downstream cytokine/proteomic assays, and (c) ambient RNA from lysed RBCs in single-cell suspensions.

**Tips:** Use PBS (optionally with 1–2 mM EDTA or 2 U/mL heparin) on ice; perfuse gently to avoid tissue edema; confirm clearance by liver blanching. If omitted, ensure robust RBC lysis and consider depleting Ly6G^+^/CD3^+^ contaminants during cleanup.

**Figure 1.**
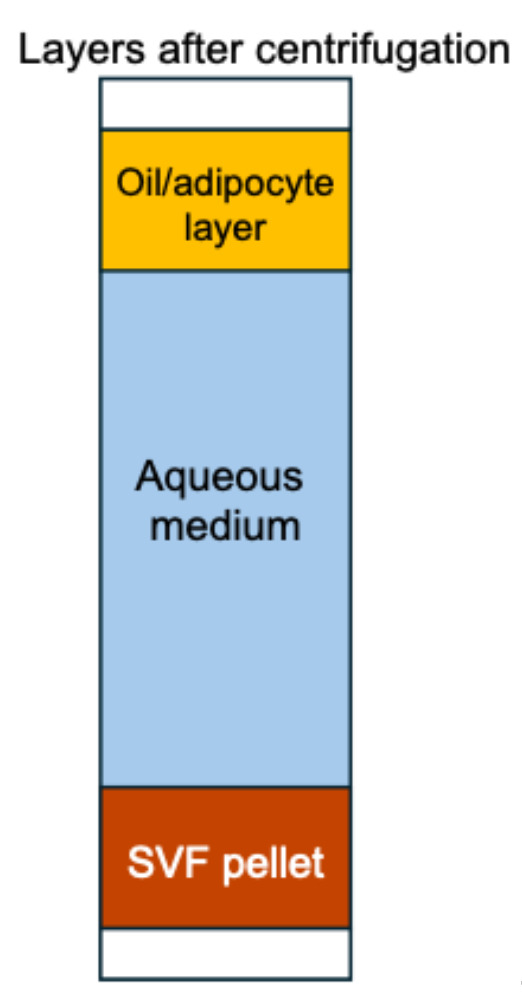
Layers formed after centrifugation of the digested adipose tissue. After centrifugation, three layers are visible: the oil/adipocyte layer floating at the top, the aqueous medium in the middle, and the stromal vascular fraction (SVF) pellet at the bottom. For downstream processing, only the SVF pellet is retained, while the top and middle layers are discarded.

**Figure 2.**
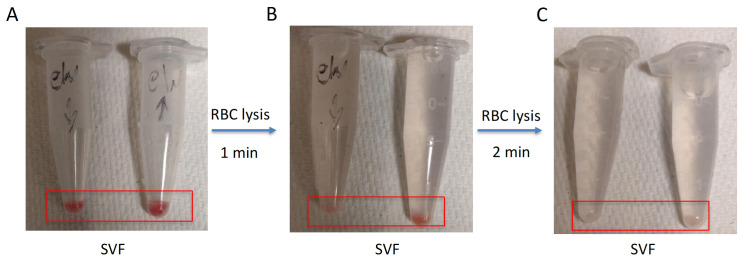
Optimization of red blood cell (RBC) lysis time during stromal vascular fraction (SVF) isolation from adipose tissue. Sequential RBC lysis was performed on SVF pellets isolated from adipose tissue. (A) Initial SVF pellets contain visible red coloration, indicating residual RBCs. (B) After a 1-min RBC lysis, partial clearance of red coloration is observed, indicating incomplete RBC removal. (C) After a 2-min RBC lysis, SVF pellets appear clear, suggesting effective removal of RBCs. Red boxes highlight the SVF pellet region in each tube for visual comparison. The red boxes highlight the SVF pellets in each condition. Handwritten tube labels are part of the original experimental images and are not required for interpreting the results.

**Figure 3.**
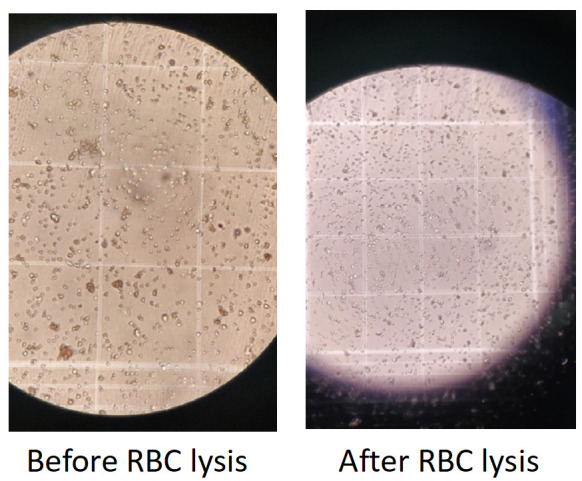
Microscopic visualization of red blood cell (RBC) removal in stromal vascular fraction (SVF) preparations. Brightfield microscopy images showing the SVF before and after RBC lysis. Left: Before RBC lysis, numerous small, dense RBCs are visible, obscuring the grid and SVF cell population. Right: After RBC lysis, RBCs are effectively removed, resulting in a clearer field with enhanced visibility of remaining SVF cells.

*Note: These images demonstrate the efficiency of the lysis step in purifying SVF for downstream applications.*

3. Excise eWAT (at least 2 g recommended) and inguinal white adipose tissue (iWAT; at least 2 g recommended) and keep in cold PBS-containing Petri dishes. While eWAT is the primary depot used for ATM isolation due to its high macrophage content in obesity, iWAT can be collected in parallel as a comparative *control* depot. Subcutaneous ATMs are less abundant and display distinct inflammatory and metabolic profiles compared to visceral ATMs, making iWAT useful for depot-specific comparisons.

4. Mince eWAT into ~1–2 mm fragments using sterile scissors in RPMI.

5. Transfer the finely chopped tissue pieces to 20 mL of Collagenase P solution (see Recipe 1) and start the digest at 37 °C for around 50 min with shaking (200 rpm). After 30 min, gently shake the tubes by hand to speed up the digestion process. Every 10 min, check the digestion status. Monitor the suspension closely and stop digestion once no large or chunky tissue fragments remain; the mixture should appear as a uniform slurry of small fragments and single cells.

6. Add 5 mL of media containing FBS to each tube and pipette well to stop the digest.

7. Filter digested tissue through a 70 μm cell strainer into a 50 mL conical tube.

8. Centrifuge at 700× *g* for 10 min at 4 °C.

9. Remove gently all the supernatants (top layer containing oil and digested fat) and the middle layer (adipocyte-containing layer). The remaining pellet is the stromal vascular fraction (reddish brown, containing RBCs) (see Figure 1).

10. Gently resuspend the SVF pellet in sterile FACS buffer by pipetting up and down until a single-cell suspension is obtained. Filter the suspension through a 70 μm strainer into a fresh 50 mL tube. Under the microscope, a heterogeneous mixture of mostly single cells (immune and stromal cells) should be visible, with minimal clumping and no large undigested fragments. Then, centrifuge at 1,000× *g* for 5 min at 4 °C.

11. Carefully remove (decant or aspirate) the supernatant without disturbing the pellet. Then, gently resuspend the pellet in 1 mL of 1× RBC lysis buffer (see step A14) and incubate at room temperature for 2 min (see Figure 2).

12. Quench lysis with 9 mL of RPMI + 2% FBS (180 μL) and centrifuge again at 1,100× *g* for 5 min. After RBC lysis, the cell pellet should be white, which depicts effective RBC lysis. Discard the supernatant and resuspend the cells in 1 mL of FACS buffer.

13. If clumps or aggregates of lysed cells are visible, pass the cells through a 70 μm strainer and keep on ice.

14. Do the cell count (optional if the cells can be judged from the pellet) (see Figure 3).

**B. Immunostaining and flow cytometry setup**

*Note: Staining steps should be performed in the dark.*

1. Take 300 μL of cell suspension in an *Unstained* labeled tube. Keep the tube on ice and do not open until the cells are transferred to a FACS Falcon tube.

*Note: Keep 40%–50% cells for the unstained tube, which is required for setting forward scatter (FSC), side scatter (SSC), and required fluorescence photomultiplier tubes (PMTs).*

2. Aliquot 1–2 × 10^6^ SVF cells per sample into FACS tubes.

3. Block Fc receptors by incubating cells with anti-CD16/CD32 antibody (1:50 dilution) in 100 μL of FACS buffer for 20 min at 4 °C. This step prevents nonspecific binding of staining antibodies to Fcγ receptors, which are highly expressed on macrophages, thereby reducing background signal and improving the specificity of flow cytometry. After incubation, spin down at 1,000× *g* for 5 min. Remove the supernatant.

4. Resuspend the pellet in 700 μL of FACS buffer and distribute 100 μL of cell suspension in the respective labeled Eppendorf.

5. Prepare antibody cocktail in FACS buffer according to the manufacturer’s recommended dilutions.

6. Add antibody cocktail to the cells and incubate for 30 min at 4 °C in the dark (see Table 1).

**Table 1.** Single-stained antibody working concentration

Name	Stock concentration	Antibody volume	Working concentration
F4/80-FITC	0.5 mg/mL	1.5 μL	0.75 μg/100 μL of million cells
CD11b-PE	0.2 mg/mL	1.25 μL	0.25 μg/100 μL of million cells
CD11C-BV421	0.2 mg/mL	1.25 μL	0.25 μg/100 μL of million cells
CD206-AF 700	0.5 mg/mL	0.5 μL	0.25 μg/100 μL of million cells
CD9-PE Dazzle594	0.2 mg/mL	1.25 μL	0.25 μg/100 μL of million cells
CD63-PE Cy7	0.2 mg/mL	2.5 μL	0.5 μg/100 μL of million cells
Trem2-APC	0.2mg/mL	5 μL	10 μg /100 μL of million cells
CD45-PerCP	0.2 mg/mL	3.75 μL	0.75 μg/100 μL of million cells

7. Wash stained cells twice with 1 mL of FACS buffer by centrifugation at 500× *g* for 5 min.

8. Resuspend cells in 300 μL of FACS buffer for acquisition.

9. Filter cells through a 35 μm mesh if necessary to avoid clogs.

10. Transfer the cells from each Eppendorf into the respective labeled 5 mL Falcon tube. Cover and keep on ice until the samples are acquired on a flow cytometer.

11. Acquire data on a flow cytometer with proper compensation controls.

*Note: Include unstained, single-color, and fluorescence-minus-one (FMO) controls for accurate gating (see Table 2).*

**Table 2.** FMO samples

**Sample name/antibodies**	F4/80-FITC	CD11b-PE	CD11C-BV421	CD206-AF 700	CD9-PE Dazzle594	CD63-PE Cy7	Trem2-APC	CD45-PerCP
FITC FMO		1.25 μL	1.25 μL	0.5 μL	1.25 μL	2.5 μL	5 μL	3.75 μL
PE FMO	1.5 μL		1.25 μL	0.5 μL	1.25 μL	2.5 μL	5 μL	3.75 μL
AF 700 FMO	1.5 μL	1.25 μL	1.25 μL		1.25 μL	2.5 μL	5 μL	3.75 μL
PE Dazzle594 FMO	1.5 μL	1.25 μL	1.25 μL	0.5 μL		2.5 μL	5 μL	3.75 μL
PE Cy7 FMO	1.5 μL	1.25 μL	1.25 μL	0.5 μL	1.25 μL		5 μL	3.75 μL
APC FMO	1.5 μL	1.25 μL	1.25 μL	0.5 μL	1.25 μL	2.5 μL		3.75 μL
PerCP FMO	1.5 μL	1.25 μL	1.25 μL	0.5 μL	1.25 μL	2.5 μL	5 μL	

**C. Gating strategy and cell sorting**

1. (Optional but recommended) Include a live/dead viability dye (e.g., Zombie Aqua, DAPI, or PI) to exclude non-viable cells. In the present study, we relied on FSC/SSC gating to remove debris and dead cells; however, for broader applications and downstream functional assays, the addition of a viability dye is strongly recommended. Although routine live/dead staining was not included in this protocol, we validated cell viability during initial optimization experiments using viability dyes. We recommend that users new to this procedure perform a viability staining step during their first trials to confirm that the protocol yields live SVF cells.

2. Exclude debris and doublets using forward scatter (FSC) and side scatter (SSC) parameters.

*Note: Autofluorescence was not used as a gating parameter in this protocol; therefore, no direct comparison with viability dyes was performed.*

3. Gate on live CD45+ leukocytes.

4. Within CD45+ cells, identify macrophages as CD11b+F4/80+.

5. Subclassify macrophages into functional subsets:

CD11c+ CD206− (classically activated/pro-inflammatory)

CD11c+ CD206+ (intermediate/dual expressing)

CD11c− CD206+ (alternatively activated)

6. To identify metabolically active ATMs, further gate CD11b+F4/80+ macrophages for:

CD9+ subset

Trem2+ subset

CD63+ subset (optional, depending on panel design)

7. For sorting:

a. Use the same gating hierarchy.

b. Sort each population into collection tubes containing RPMI + 10% FBS.

c. Keep cells at 4 °C and proceed immediately with downstream analysis (e.g., RNA isolation).

**Critical:** Ensure the sorter is well-calibrated and that collection tubes are pre-coated with FBS. To pre-coat, add ~0.5–1 mL of FBS to each tube, gently swirl to cover the inner surface, and then discard the excess. This leaves a thin protein layer on the tube walls, which helps prevent cell adhesion and reduces cell loss during collection.

## Data analysis

**Figure 4.**
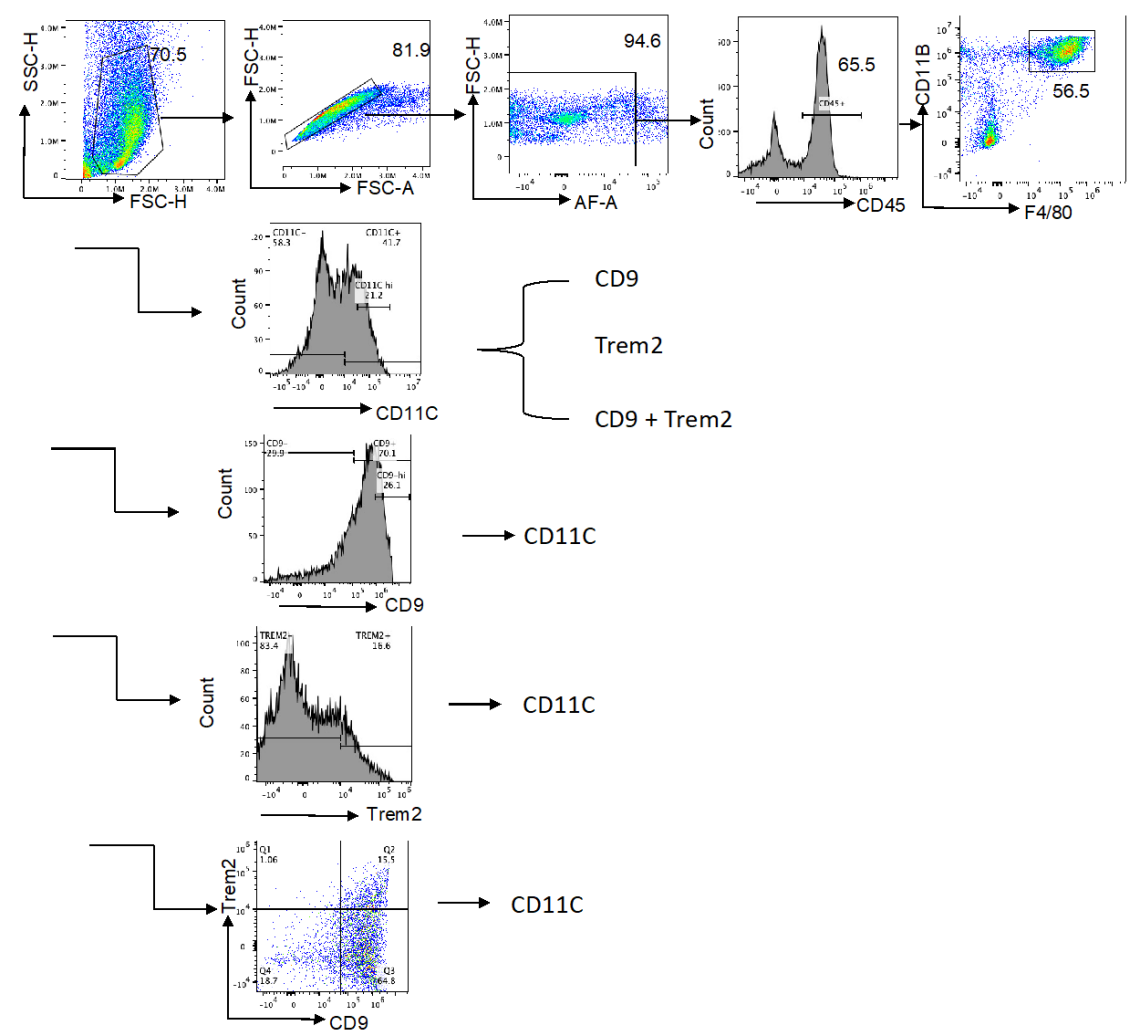
Flow cytometry gating strategy for identification of macrophage subsets in the stromal vascular fraction (SVF). Representative plots illustrate the sequential gating of SVF cells, including singlet and CD45 populations, macrophages (CD11bF4/80), and downstream subsets (CD11c, CD206, CD9, TREM2). Gates are indicated to show the strategy used for analysis and sorting. This figure is intended as a schematic overview of the gating hierarchy; detailed quantitative data and subset percentages are reported in Wu et al. [16].

1. Analyze acquired flow cytometry data using FlowJo v10 or equivalent software.

2. Apply compensation matrices to correct spectral overlap between fluorophores.

3. Use sequential gating to identify ATM populations as described in Section C (see Figure 4).

4. Quantify the percentage and absolute count (if counting beads are used) of:

CD11c+ ATMs

CD206+ ATMs

CD9+ and Trem2+ metabolically active ATMs

5. Export gated population statistics for further statistical analysis using GraphPad Prism.

6. For sorted cells, extract total RNA and perform downstream gene expression analysis using RT-qPCR or RNA-seq.

Branch 1 (Analysis only) → Flow cytometry → Data analysis.

Branch 2 (Sorting path) → Cell sorting → RNA isolation → PCR/RNA-seq.

7. Normalize gene expression data using appropriate housekeeping genes (*Gapdh*).

8. Perform statistical testing using unpaired t-tests or ANOVA with post hoc corrections as appropriate. Present data as mean ± SEM and specify the number of biological replicates.

## Validation of protocol

This protocol has been rigorously validated and reproduced across multiple biological replicates in the context of a diet-induced obesity (DIO) model. Sorted ATM subsets were subjected to RT-qPCR, confirming distinct gene expression profiles consistent with pro-inflammatory and metabolically activated phenotypes. In particular:

- CD11c+ ATMs exhibited elevated expression of *Il1b* and *Tnfa* in high-fat diet (HFD)-induced compared to lean mice.
- CD9+ and Trem2+ ATMs showed increased expression of lipid-handling and lysosomal genes, including *Fabp4* and *Lipa* in HFD-induced compared to lean mice.
- Pharmacological inhibition of IRE1 with STF-083010 significantly reduced the abundance of pro-inflammatory CD11c+ and metabolically active CD9+ ATMs.
  This protocol (or parts of it) has been used and validated in the following research article:

- Wu et al. [16]. Targeting IRE1 improves insulin sensitivity and thermogenesis and suppresses metabolically active adipose tissue macrophages in male obese mice. *eLife*.

## General notes and troubleshooting

**General notes**

1. Ensure consistent timing for tissue digestion to avoid over- or under-digestion, which can compromise yield and cell surface marker integrity.

2. Maintain samples on ice and minimize light exposure during antibody staining to preserve fluorescence signal integrity.

3. It is recommended to pool SVF from 2–3 mice per sample when sorting rare ATM populations (e.g., Trem2+).

4. Adjust antibody panels based on instrument configuration and availability of detection channels.

5. If using this protocol in female mice or different fat depots (e.g., subcutaneous), optimize digestion and gating parameters accordingly.

**Troubleshooting**

**Problem 1:** Low cell yield after digestion.

Possible cause: Incomplete digestion or poor tissue mincing.

Solution: Ensure uniform mincing of adipose tissue and verify collagenase activity. Extend digestion time slightly if needed.

**Problem 2:** High background signal or poor resolution between subsets.

Possible cause: Nonspecific antibody binding or suboptimal staining conditions.

Solution: Include Fc block and titrate antibodies for an optimal signal-to-noise ratio.

**Problem 3:** Cell clumping during acquisition or sorting.

Possible cause: Incomplete filtration or high cell concentration.

Solution: Filter cells immediately before use and dilute in FACS buffer with EDTA to prevent aggregation.
